# Neuromodulatory Control of a Goal-Directed Decision

**DOI:** 10.1371/journal.pone.0102240

**Published:** 2014-07-21

**Authors:** Keiko Hirayama, Leonid L. Moroz, Nathan G. Hatcher, Rhanor Gillette

**Affiliations:** 1 Department of Molecular & Integrative Physiology, University of Illinois, Urbana, Illinois, United States of America; 2 The Neuroscience Program, University of Illinois, Urbana, Illinois, United States of America; Mount Sinai School of Medicine, United States of America

## Abstract

Many cost-benefit decisions reduce to simple choices between approach or avoidance (or active disregard) to salient stimuli. Physiologically, critical factors in such decisions are modulators of the homeostatic neural networks that bias decision processes from moment to moment. For the predatory sea-slug *Pleurobranchaea*, serotonin (5-HT) is an intrinsic modulatory promoter of general arousal and feeding. We correlated 5-HT actions on appetitive state with its effects on the approach-avoidance decision in *Pleurobranchaea*. 5-HT and its precursor 5-hydroxytryptophan (5-HTP) augmented general arousal state and reduced feeding thresholds in intact animals. Moreover, 5-HT switched the turn response to chemosensory stimulation from avoidance to orienting in many animals. In isolated CNSs, bath application of 5-HT both stimulated activity in the feeding motor network and switched the fictive turn response to unilateral sensory nerve stimulation from avoidance to orienting. Previously, it was shown that increasing excitation state of the feeding network reversibly switched the turn motor network response from avoidance to orienting, and that 5-HT levels vary inversely with nutritional state. A simple model posits a critical role for 5-HT in control of the turn network response by corollary output of the feeding network. In it, 5-HT acts as an intrinsic neuromodulatory factor coupled to nutritional status and regulates approach-avoidance via the excitation state of the feeding network. Thus, the neuromodulator is a key organizing element in behavioral choice of approach or avoidance through its actions in promoting appetitive state, in large part via the homeostatic feeding network.

## Introduction

Major decisions in foraging behavior concern the approach or avoidance of salient stimuli in the environment. Such decisions are generally made on a cost-benefit basis where decision is informed by the moment-to-moment integration of sensation, internal state and memory. Foraging behavior is regulated by animal arousal state and appetite, themselves organized by neuromodulatory systems that regulate homeostatic neuronal networks and the sensory and motor ensembles that serve them. Documenting and testing the roles of neuromodulation in decision are significant to understanding and modeling the neural and behavioral economics of foraging.

The opisthobranch and pulmonate gastropod molluscs offer model organisms in which the neuromodulatory players in arousal and appetitive state can be addressed directly. For these molluscs serotonin (5-HT) is the most prominent neuromodulator yet found to affect arousal and appetitive state through its stimulatory effects on behavior and neural activity when applied to intact animals or to the isolated CNS [1]–[4]. In particular, Palovcik et al. [5] showed that injected 5-HT promoted arousal and appetitive state in terms of general activity and reduced sensory thresholds and latencies for eliciting feeding behaviors in the predatory sea-slug *Pleurobranchaea*. 5-HT is a central, and possibly the main, neuromodulatory factor regulating arousal and appetitive state through its likely roles in mediating circadian activity rhythms [6], [7] and satiation [8].

Appetitive state, the readiness for expressing appetitive behavior, represents the integration of sensation, internal state, and memory. Hirayama and Gillette [9] found that appetitive state in *Pleurobranchaea* is expressed directly in the excitatory state of the feeding motor network, which sums effects of sensation, satiety and learning into the intensity and configuration of neuronal activity. It was of particular interest to find that the excitatory state of the feeding network controlled the switch between approach and avoidance behavior, converting avoidance turn responses to orienting with increasing excitation. A potential significant role for 5-HT in the decision process was suggested by its actions as an intrinsic modulator of the feeding CPG network and diverse other neuronal circuits [10], [11].

We addressed how 5-HT might influence the choice of approach-avoidance in behavioral choice of intact animals and in the fictive turn responses of their isolated CNSs. The results are consistent with the role of 5-HT as a potent neuromodulator that promotes the excitation of the feeding motor network. The enhanced excitatory state biased the switching of the motor output of the turn network from avoidance to orienting responses to sensory stimuli. These results add to the known role of 5-HT as a central organizing factor in gastropod behavior and provide a novel example of the neuromodulatory regulation of a homeostatic decision.

## Materials and Methods

Specimens of *Pleurobranchaea californica*, 80–1000 ml volume, were obtained by trawl or trapping through Sea Life Supply, Sand City, CA and Monterey Abalone, Inc., Monterey, CA. and maintained in artificial seawater at 12–13°C until use.

Appetitive state, measured as behavioral readiness-to-feed, in *Pleurobranchaea* is controlled by sensation, nutritional state, learning, reproductive condition and health. Readiness-to-feed here is quantified in terms of feeding thresholds measured as the minimal concentrations of appetitive stimuli to elicit proboscis extension and active biting. Feeding thresholds were measured as previously described [12], [13] in response to squid homogenate or betaine (trimethylglycine; Sigma-Aldrich) solutions in seawater with 10 mM MOPS buffer (3-(N-morpholino)propanesulfonic acid) at pH 8.0, applied in 1.5 ml volumes to the oral veil with a hand-held Pasteur pipette over 10 seconds in a series of ascending concentrations in ten-fold steps. Squid homogenate was prepared as a fresh 1∶1 squid-seawater cheesecloth filtrate, assigned a value of 10^0^, and ten-fold dilutions were prepared down to 10^−6^. Betaine was freshly prepared as 10^−1^ M in seawater and dilutions were prepared down to 10^−6^ M. Parameters recorded were those concentrations at which animals showed proboscis extension and biting. When specimens failed to respond to the highest concentration (10^0^ squid homogenate or 10^−1^ M betaine) the next highest values, 10^1^ or 10^0^ for squid and betaine, respectively, were assigned. Tests began with a control sea-water application assigned a value of 10^−7^. These conventions assign conservative finite values to essentially infinitely high or low thresholds. Threshold data were analyzed and presented as the logarithms of the dilutions; thus, 10^−1^ is −1.0 and so on.

Results were analyzed using non-parametric methods for the non-Gaussian distribution of the data. Friedman's non-parametric repeated measures ANOVA was used to detect differences in treatments across multiple tests. Wilcoxon matched-pairs signed-ranks tests were used to compare repeated measurements to assess whether control or experimental population mean ranks differed with time. Kruskal–Wallis ANOVA was used to compare groups of unequal size. The Mann-Whitney test was used to test for significant differences in control vs. experimental thresholds. Population thresholds are presented as medians, and errors are presented as interquartile range (+/− IQR). Data are presented in box and whisker charts where the ends of the whisker are set at 1.5*IQR above the third quartile (Q3) and 1.5*IQR below the first quartile (Q1). When Minimum or Maximum values are outside this range, they are shown as outliers.

Taurine for behavioral testing was made as a 10^−2^ M solution in artificial sea-water (pH 8.0). 5-hydroxytryptamine creatine sulfate (5-HT; Sigma-Aldrich) solutions were prepared for hemocoele injection as 1 mM in artificial saline (below) and as 2.5, 5 and 50 µM solutions for bath application to isolated CNSs. The 5-HT precursor 5-hydroxytryptophan (5-HTP; Sigma-Aldrich) was prepared for hemocoele injections as a 1 mM solution in artificial saline. For estimation of final dilutions after injections, animal volumes were measured prior to injections and hemolymph volumes were estimated at 65% of total volume. Animals were satiated in some experiments by feeding strips of squid flesh until they stopped eating, having consumed quantities of 10–35% body weight.

For electrophysiological recordings animals were anesthetized by cooling to 4°C. CNSs, consisting of interconnected cerebropleural, pedal, visceral and buccal ganglia, were dissected out and pinned in a Sylgard dish under artificial saline (in mM) 460 NaCl, 10 KCl, 25 MgCl_2_, 25 MgSO_4_, 10 CaCl_2_, and 10 MOPS buffer at pH 7.5 and 12–13°C. Suction electrodes recorded activity in buccal ganglion motor nerve root 3 (R3), which displays both rhythmic and non-rhythmic output of the feeding central motor pattern generator, and from the bilateral lateral body wall nerves (LBWNs) which are motor outputs for the turn network.

Fictive turns were induced by brief stimulation of one of the bilateral pair of large oral veil nerves (LOVNs; 15 Hz, 2 msec pulse duration; [14]). Data were captured and analyzed with Chart 5 Pro (AD Instruments). Spikes were counted at threshold levels above spontaneous noise and spike frequencies were normalized to counts for 20 seconds prior to the stimulus event and plotted in 2–3 second bins. Fictive turn events were assessed by comparing mean spike frequencies in LBWNs [9], [14] and by comparing the ratios of relative spike frequencies across bins in the ipsilateral vs. contralateral turn nerves. Kruskal–Wallis ANOVA was used to assess variations across ratio medians, and the two-tailed Dunn's Multiple Comparisons Test was used to compare ratio differences between control and experimental (5-HT) conditions. P values were calculated by comparing the spike counts in ipsilateral and contralateral LBWNs for 30 seconds from the first steep inflection following the initial peak. The initial peak corresponds to a fictive withdrawal preceding the turn, as in intact animals [14]. Criteria for assigning “fictive avoidance” vs. “fictive orienting” to LBWNs' activities were significant differences of at least p<0.05 for bilateral spike counts [9], and values for ratios of ipsilateral/contralateral nerves spike frequencies of less than or greater than 1.0, respectively. To assess effects of 5-HT on turn direction, measured volumes of 1 mM 5-HT in saline were added to the recording chamber by pipette and gently mixed to final concentrations of 5–50 µM.

## Results

Orienting and avoidance turns have been well described in intact animals [14], [15]. Strong avoidance turn responses to transient noxious stimuli are relatively stereotypic, mediated by contraction of longitudinal body wall muscles and body flexion away from the stimulus to 45–250°, accompanied by suppression of locomotion and feeding, and are generally complete in 30–40 seconds. Orienting turns tend to be of smaller angles, slower, and do not always interrupt locomotion. Turn stimuli induce a transient withdrawal (around 10 seconds) preceding avoidance turns and sometimes orienting turns as well. These characters are reflected in the fictive behavior of the isolated CNS.

### 5-HT Reduces Feeding Thresholds and Biases Turn Choice to Orienting in Intact Animals

**Figure 1:**
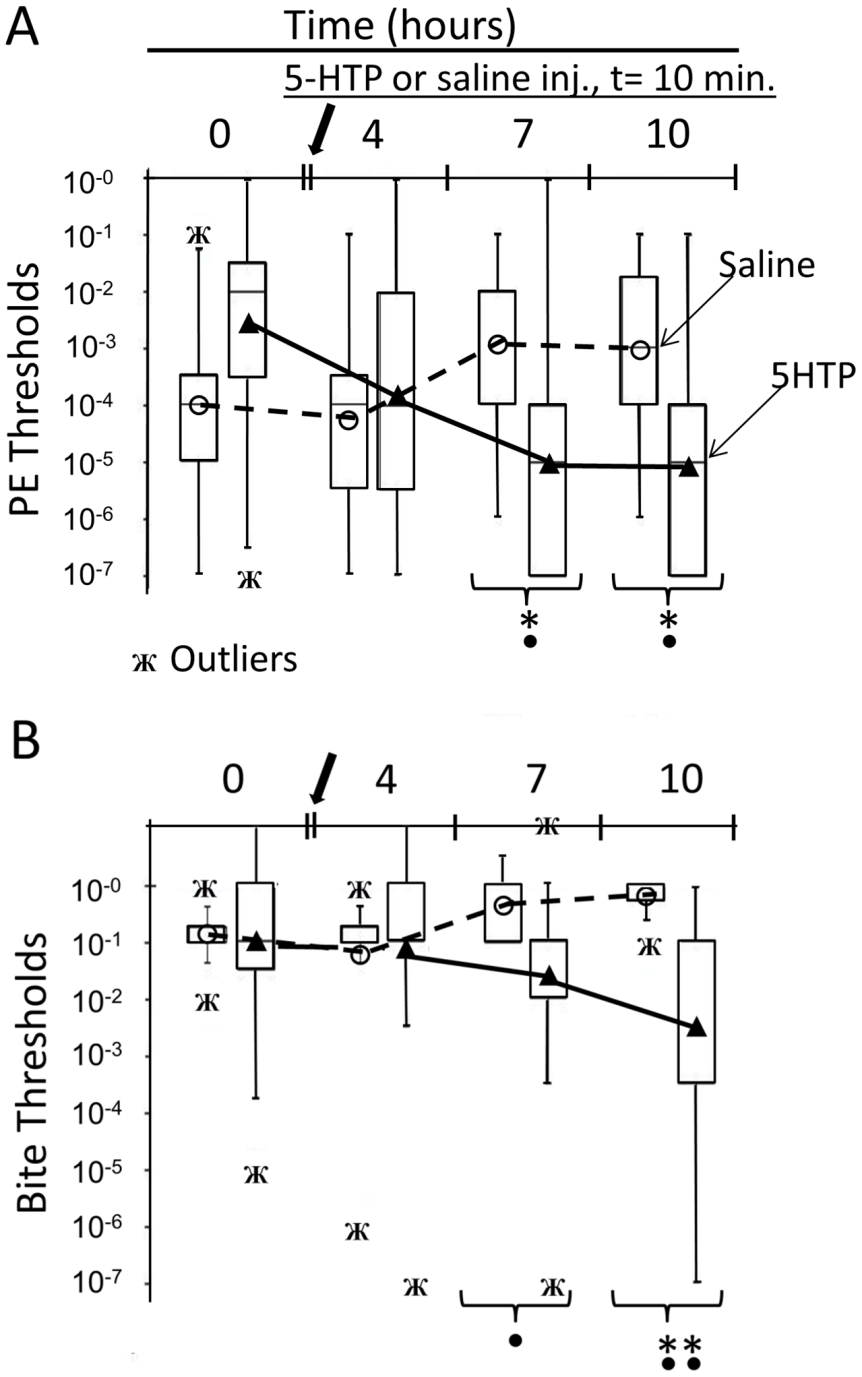
5-HTP injection reduced thresholds for both proboscis extension (A) and biting (B). Following initial threshold measures at t = 0 with squid homogenate dilutions, animals were injected with 5-HTP (50 µM estimated internal dilution) at t = 10 min. (○, group mean experimentals; N = 15) or isotonic saline (▴, group mean controls; N = 8) and measured at intervals over 10 hours. Proboscis extension (PE) and bite thresholds differed significantly over trials in the 5-HTP injected groups (Friedman's non-parametric repeated measures ANOVA, χ2 = 21.744 (PE), χ2 = 12.830 (Bite), p<0.0001 and p<0.005, respectively). Experimentals' PE thresholds were significantly reduced starting at 7 and 10 hours (Wilcoxon matched-pairs signed-ranks tests: W = −101, *p<0.0005). Bite thresholds were significantly reduced at 10 hours (**p<0.0001). 5-HTP injected experimentals differed over time from saline injected controls (Kruskal-Wallis Non-parametric ANOVA, H = 19.315 (PE), H = 14.838 (Bite), p<0.01 and p<0.04 for PE and Bite thresholds, respectively). Threshold differences between experimentals and controls were significant at 7 and 10 hours (Mann-Whitney tests, ^•^p<0.02). Bite threshold increased over the experiment in saline injected controls (Friedman's non-parametric repeated measures ANOVA, χ2 = 8.882, p<0.02), suggesting handling effects [16], but the individual increases were non-significant.

Injecting animals with the 5-HT precursor 5-HTP (50 µM estimated internal dilution) significantly reduced feeding thresholds for proboscis extension and biting at 7 hours post-injection; measured effects peaked at 10 hours post-injection (Fig. 1). The appearance of spontaneous locomotor activity, spontaneous local mantle contractures, handling-induced biting and escape swimming were consistent with a state of general arousal. Our field observations suggest that these features are also characteristic of naturally very hungry animals freshly captured in trawls. In contrast, saline-injected control animals showed slightly elevated feeding thresholds, which are characteristic of handling effects [16].

Subsequently, we tested effects of 5-HTP on satiation. Eight animals were fed to satiation, raising averaged biting threshold 100-fold. Injecting 4 animals with 5-HTP lowered thresholds to pre-satiation values (p<0.03; Fishers Exact test) and increased general arousal state as above, although crops and stomachs remained visibly full of squid, as seen through the translucent mantle. Saline-injected controls retained high thresholds.

**Figure 2:**
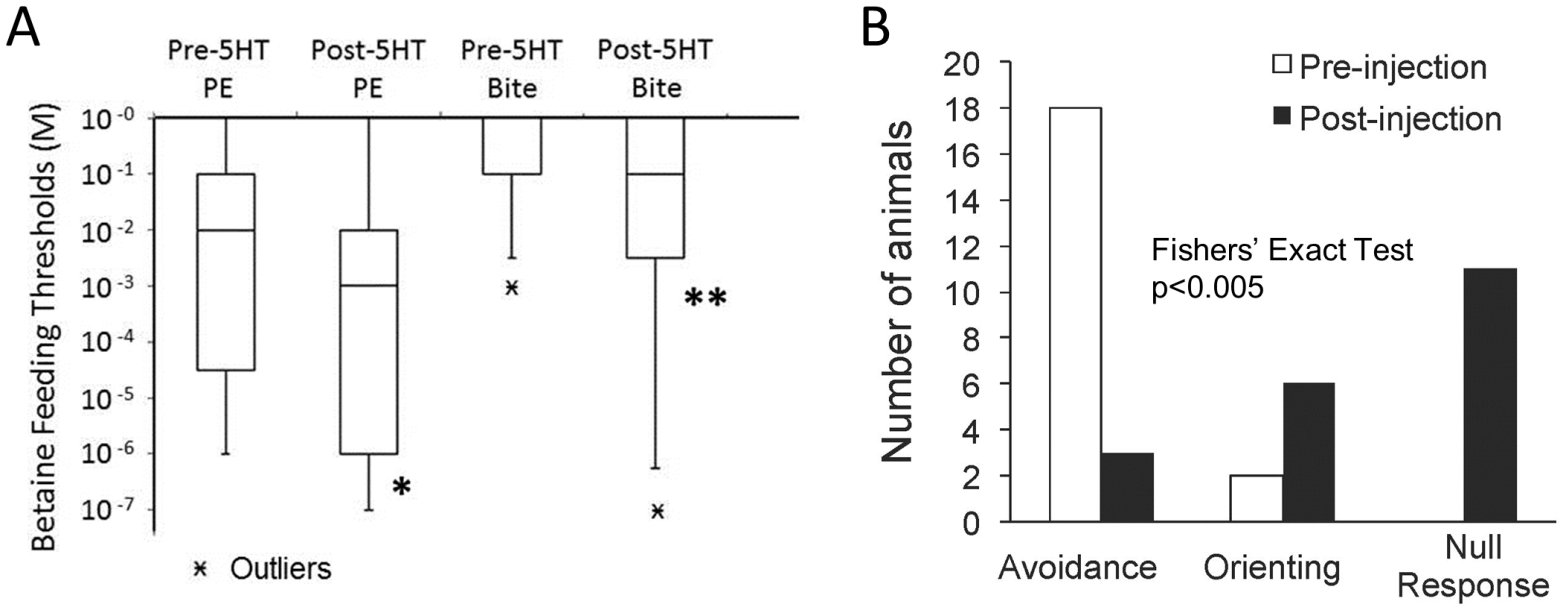
5-HT injections reduced feeding thresholds, suppressed avoidance, and promoted orienting turn responses to a noxious stimulus in intact animals. A. Injection of 23 animals with 5-HT (1–2.5 µM final hemolymph dilution) significantly reduced median feeding thresholds to betaine measured 12 minutes later (Wilcoxon matched-pairs signed-ranks tests, PE: W = −28, *p<0.02; Bite: W = −36, **p<0.01). Control saline injections were ineffective. B. Responses to noxious unilateral taurine application (10^−2^ M) to the tentacle/oral veil were compared before and after 5-HT injection to an estimated final hemolymph concentration of 2.5 µM. Among 20 animals tested, 18 initially showed avoidance and 2 animals showed orienting responses to taurine application. 12 minutes after 5-HT injection, avoidance was replaced by null responses in 11 animals and orienting turns were observed in 6 (p<0.005, Fishers' Exact Test); only 3 animals continued to show avoidance.

Injections of 5-HT caused behavioral effects similar to the 5-HTP precursor, but with much shorter latency. Twenty-three animals were injected with sufficient 1 mM 5-HT for an estimated hemolymph dilution of 2.5 µM. This treatment lowered the averaged thresholds for inducing proboscis extension and biting with betaine by 27- and 15-fold, respectively, and significantly reduced median biting thresholds when animals were tested at 12 minutes post-injection (Fig. 2A). General behavioral arousal, as seen with 5-HTP, also appeared quickly and effects lasted over 40 minutes. Control injections of saline alone given to the animals on the days previous and following 5-HT injection caused no significant changes. Injections of a much larger amount of 5-HT (50 µM final dilution) into 15 different animals over-dosed them into 40–60 minutes of seeming locomotor paralysis accompanied by spasmodic waves of local mantle contracture, transient (5–10 minutes) penis eversion and non-responsiveness to mechanical and appetent chemical sensory inputs. Such effects recall the inverted U-shaped function typical of the relation for performance and arousal [17], where performance is diminished at higher arousal states. On eventual recovery of sensory responsiveness and locomotor ability, animals entered a state of general activity as above for an hour or more. Thus 5-HT stimulated arousal state in general, and readiness to feed in particular.

5-HT reduces avoidance and promotes appetitive behavior to a mildly noxious stimulus like that observed in untreated animals with high readiness to feed.Turning responses to taurine were also altered at 12 min. after 5-HT injection (2.5 µM final hemolymph concentration). Taurine is a deterrent stimulus that induces skin acid secretion, which itself is aversive to the animal [15]. Unilateral application of 10^−2^ M taurine to the oral veil normally induces avoidance responses in animals with mid- to high-level feeding thresholds. However, in animals with lower feeding thresholds, taurine, acidified sea-water and moderate mechanical stimuli induce feeding attack [12]. We tested 20 animals with higher feeding thresholds (biting at or above 10^−1^ M betaine) and found that 18 animals avoided the taurine stimulus and 2 animals showed orienting responses. 12 minutes after the 5-HT injection, turn responses ceased in 11 subjects, orienting turns were elicited in 6, and only 3 animals continued to show avoidance turns (Fig. 2B).

### 5-HT Promotes Fictive Orienting Turn Choice and Excitation in the Feeding Network of Isolated CNSs

**Figure 3:**
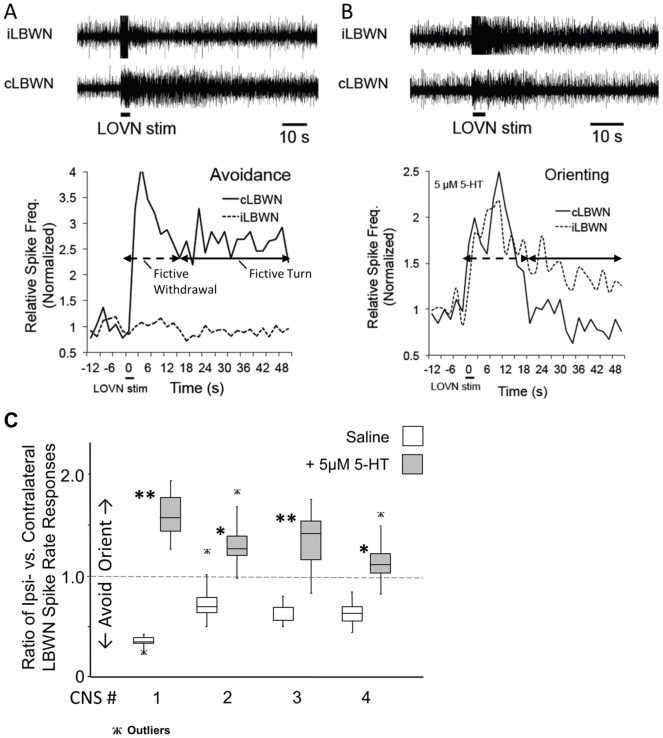
5-HT altered fictive turn preference from avoidance to orienting. Application of 5 µM 5-HT to isolated CNS from each of 4 donors changed fictive turn preference from avoidance to orienting. The figure shows a fictive avoidance turn (A) changed to fictive orienting (B) within 5 minutes following 5-HT addition. Fictive turn direction is shown in differing relative spike rates of the LBWNs following stimulation of the ipsilateral LOVN (short horizontal bar at bottom) [9]. Fictive turns (solid double arrows) are measured following transient fictive withdrawal responses (dashed double arrows). C. Box and whiskers plot of ratios of spike frequencies for the LBWNs calculated across bins to assess orienting vs. avoidance turns in control vs. 5-HT conditions. Variations across ratio medians were significant (Kruskal–Wallis ANOVA; p<0.0001). Ratios of spike frequencies in 5-HT were significantly different from controls (two-tailed Dunn's Multiple Comparisons Test; *p<0.01; **p<0.001; Table 1).

Choice of fictive turn response, avoidance or orienting, in isolated CNSs responding to unilateral sensory nerve stimuli was previously found to correlate with donor animals' feeding thresholds, and to be regulated by the excitation state of the feeding network [9]. Thus, we assayed effects of 5-HT on fictive turn choice in CNSs from four donor animals with high feeding thresholds (betaine thresholds 10^−1^ M and above) that responded initially with fictive avoidance to unilateral stimulation of the sensory LOVN. Tested within 5 minutes after bath application of 5 µM 5-HT, all four CNSs showed fictive orienting to the LOVN stimulation (Fig. 3, Table 1), significant at p<0.03 (two-sided Fisher's Exact Test). Increased overall spiking activities were observed in both turn and feeding motor nerves soon after 5-HT application and gradually declined over 10–20 minutes.

**Table 1:** Ratios of relative spike rate responses of contralateral vs. ipsilateral turn nerves to unilateral LOVN stimulation.

Experiment	Initial Avoidance Response: ipsilateral/contralateral LBWN spike frequencies	5-HT Induced Orienting Response: ipsilateral/contralateral LBWN spike frequencies	Two-Sided Fisher's Exact Test: Significance of the 5-HT Effect
1	0.349	1.592**	P = 0.0286
2	0.739	1.328*	
3	0.616	1.350**	
4	0.628	1.140*	

Dunn's Multiple Comparison Test; *p<0.01, **p<0.001. Avoidance turns are characterized by ratios <1.0, and orienting turns by ratios >1.0. Fisher's Exact Test supports a significant effect of 5-HT in changing avoidance responses to orienting in the four experiments of Figure 3.

**Figure 4:**
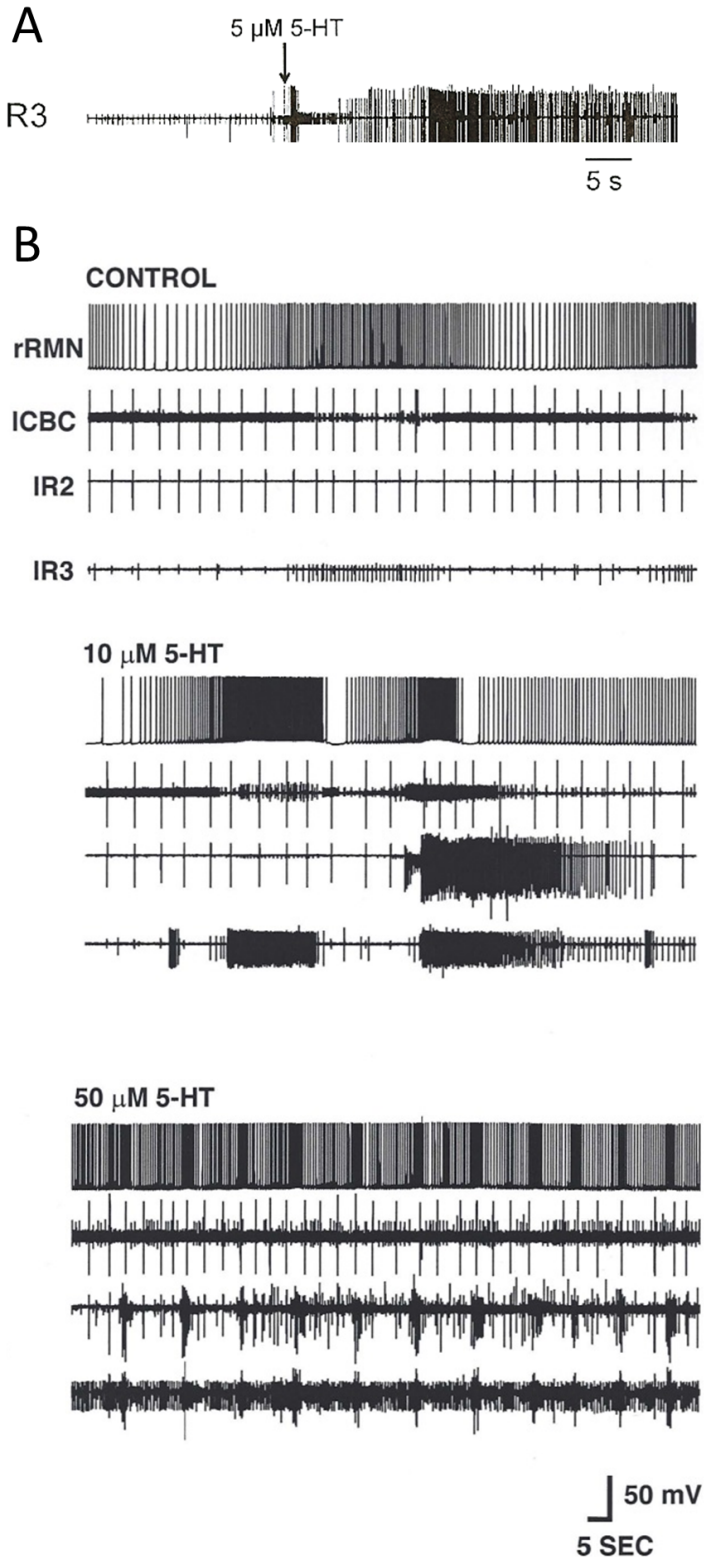
5-HT stimulates general excitation state of the feeding motor network in isolated CNSs. Similar stimulation of the feeding network by driving command neurons or gastroesophageal stimulation converts fictive avoidance turn responses to orienting [9]. A. 5 µM 5-HT induces spike activity in nerve root 3 (R3) of the buccal ganglion. B. A separate experiment in which sequential applications of 10 and 50 µM 5-HT show dose-dependence of induced spiking activity recorded intracellularly in a right retraction phase buccal motorneuron (rRMN) and extracellularly in a left cerebrobuccal connective (lCBC), and left nerve roots 2 and 3 (lR2, lR3).

It was shown that artificially increasing the excitatory state of the feeding network can cause the switch from fictive avoidance to orienting turns [9]. Thus, we tested effects of 5-HT on the feeding network. No robust differences were observed in the sensitivity to 5 µM 5-HT in CNSs from 5 high- and 5 low-threshold animals (not shown). However, 5-HT had dose-dependent excitatory effects on the feeding network at concentrations of 5–50 µM, with lesser and more variable effects at the lower concentrations (Fig. 4).

## Discussion

These observations directly demonstrate regulation of a homeostatic (feeding) network by an intrinsic neuromodulatory factor, thereby directing goal-seeking behavior. The results support a central role for 5-HT in regulating appetitive state in the molluscan CNS, and they extend the neuromodulator's known functions beyond general arousal mechanisms and plasticity to an elemental, value-based decision: the goal-directed turn.

5-HT is a well-recognized regulator of appetitive state and arousal in lophotrochozoan (mollusc and leech) model systems. This is well documented across molluscan species: for the sea-butterfly *Clione limacina* [18], the pond snails *Helisoma trivolvis* [19], *Lymnaea stagnalis* [20], and *Planorbis corneus* [21], the sea-hare *Aplysia californica* [22], and the side-gilled sea-slug *Pleurobranchaea californica* [5], [8]. In particular, the work of C. M. Lent and collaborators [23], [24] indicated that satiation-related changes in levels of 5-HT might modulate homeostatic decision in the leech, where increasing CNS levels would promote prey search, attack and feeding, while decreasing 5-HT levels accompanied quiescence and crypsis. Similar observations in *Pleurobranchaea* [8] suggested a role for 5-HT in mediating cost-benefit decisions for approach and avoidance, and spurred our investigations.

The main findings of this study were three: 1) Exogenous 5-HT and its 5-HTP precursor enhanced expressions of appetitive state in both the intact animal and the isolated CNS; 2) 5-HT increased overall excitation state in the feeding motor network; and 3) Avoidance turns expressed in intact animals and fictively in isolated CNS could be converted to orienting by exogenous 5-HT, paralleling expression of appetitive state in feeding thresholds of intact animals and spontaneous activity in the feeding motor network.

The effects of 5-HT as a global modulator of behavioral state were confirmed and were extended to show that 5-HTP, the precursor of 5-HT, had stimulatory effects in the intact animal similar to native 5-HT. The action of 5-HTP in enhanced stimulation of molluscan neuronal circuitry has been related to increased presynaptic 5-HT content and release [25]–[27].

Behavioral effects of 5-HT were further extended to approach-avoidance responses to a noxious stimulus, taurine. Taurine at concentrations of 10^−5^ to 10^−2^ M induces acid secretion in the skin of *Pleurobranchaea*, which is normally a defensive chemical response that also acts as an auto-irritant to potentiate aversive behavior [15]. However, animals with lower feeding thresholds can respond to noxious stimuli like taurine and acidified sea-water with appetitive behavior [12]. This may be an adaptive behavioral response, where mildly painful stimuli reinforce attack behavior in the hungry predators to deal with struggling prey [12]. 5-HT injections changed taurine-avoidance responses to either orienting or null responses in a significant number of subjects tested, showing a stimulating action of 5-HT on appetitive state.

5-HT stimulates the excitation state of sensory pathways and multiple motor networks in the molluscan nervous system, among them the feeding motor network. In particular, the excitation state of the feeding motor network directs the approach-avoidance decision of the goal-directed turn [9], and these results connect the actions of 5-HT in the feeding motor network to regulation of the turn motor network in behavioral choice. The conversion of fictive turn decision from avoidance to orienting by 5-HT is consistent with the neuromodulator's action in promoting feeding network excitation, and resembles the effects of direct stimulation of the feeding CPG by driving feeding command neurons or feeding nerves [9].

**Figure 5:**
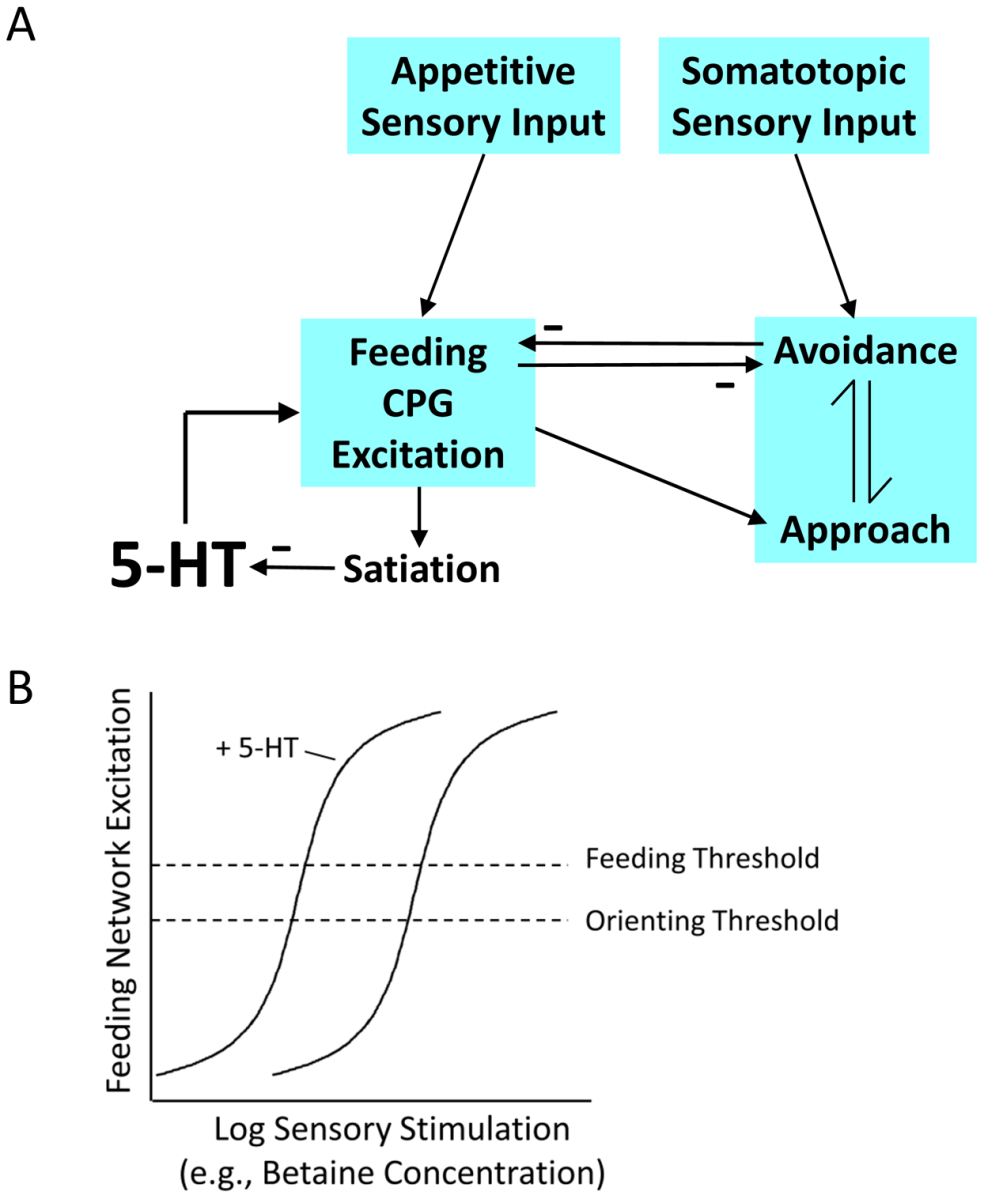
A model for regulation of appetitive state and behavioral choice by satiation via serotonin. A. The model builds on the relation that the default turn response to somatotopically mapped stimuli from the oral veil is avoidance, and that corollary outputs of the feeding network can change this response to orienting. The resting excitation state of the feeding motor network is set by endogenous 5-HT level, which is an inverse function of satiation state. Thus, network excitation state sums effects of satiation and appetitive sensory inputs (including effects of learned values of different prey odors). At increasing levels of feeding network excitation the turn response is switched from avoidance to approach. B. Increasing levels of 5-HT, through decline of satiation or by exogenous addition, increase feeding CPG excitation and thereby reduce the sensory thresholds for orienting and active feeding.

A model for the role of 5-HT in approach-avoidance choice is based on regulation of basal excitation state in the feeding motor network (Fig. 5A). The model combines the present results with previous findings that resting excitation state in the feeding network of the isolated CNS is proportionate to donors' feeding thresholds, where excitation state is significantly higher in animals with lower thresholds [9]. The turn network responds by default to unilateral, somatotopically mapped sensory input from the oral veil [28] with avoidance turns, and increasing feeding network activity switches turn responses to orienting [9]. The model posits that variations in endogenous 5-HT in the feeding network regulate excitability, based on findings that 5-HT content in serotonergic neurons of the feeding motor network varies inversely with satiation state [8]. Thus, higher levels of endogenous 5-HT in the feeding networks of more ready-to-feed animals may underlie their enhanced appetitive states, lower feeding thresholds, and their consequent biases toward orienting turns (Fig. 5B).

We speculate that endogenous 5-HT acts in the feeding network within a neuromodulatory positive feedback loop, where temporal decline in primary satiation factors (e.g., gut stretch) would result in a sharpened appetite due to the excitatory action of recovering 5-HT activity. Thus, in modulating gastropod appetitive state, 5-HT may act much as the hypothalamic orexin/hypocretin peptides do in mammals [29], and is similarly regulated by more primary satiation factors. In gastropods, gut stretch is the primary satiation factor, and acts in *Pleurobranchaea* to bias the rhythmic radular protraction/retraction cycle of the feeding motor CPG towards the retraction phase, thereby suppressing feeding (reviewed in ref. 11). Possibly, satiation-induced decrease in 5-HT levels in modulatory feeding neurons might be caused by use-dependent effects of decreased activity in the feeding network. This suggestion is supported by an early observation of an increase with depolarization of a 5-HT-like intracellular voltammetry signal in a homologous neuron of the gastropod *Aplysia* [30]. Otherwise, an effect of some other, as yet unidentified, satiation factor could mediate effects on 5-HT levels.

Serotonergic neurons in the gastropod nervous system are embedded in motor networks as intrinsic neuromodulatory elements and they are loosely coupled across the CNS to form a distributed serotonergic network important in behavioral arousal, sensitization, and learning [11], [27], [31], [32]. In the vertebrates, 5-HT has similar neuromodulatory roles in excitatory arousal of sensory and motor circuits, consistent with evolutionary conservation of function. However, its vertebrate arousal functions are secondary and subservient to the hypothalamic neuropeptide orexin/hypocretin and a neuromodulatory network of other peptides in regulating arousal and appetitive state. This relationship has been related to a relative lack of communication between the CNS and nutritional stores in those lophotrochozoans that have been studied [11], unlike the complex regulation of appetite in vertebrates by hormonal communication with CNS from nutritional stores (fat, glucose and glycogen) and the gut via the hypothalamic peptidergic network [33]. Thus, animals like *Pleurobranchaea* with simpler behavioral economies use minimal systems of neuromodulatory controls of appetitive state and cost-benefit approach/avoidance decisions that may well resemble those built upon in vertebrate evolution.

*Pleurobranchaea* readily learns the values of environmental stimuli by experience [16], and the effects of learning are integrated into appetitive state along with sensation and satiation in the feeding network [9], [34], [35]. Thus, approach/avoidance decision in the directed turn is an elemental, goal-directed cognitive choice that embodies expected utility and risk. The neuromodulatory role of 5-HT as a central regulator of homeostatic decision offers an example for homeostatic regulation of goal-directed decision that can be readily pursued in this and similar systems.
